# Study on the kinetics and influence of feline platelet aggregation and deaggregation

**DOI:** 10.1186/s12917-015-0590-7

**Published:** 2015-11-05

**Authors:** Barbara Riond, Andrea Katharina Waßmuth, Sonja Hartnack, Regina Hofmann-Lehmann, Hans Lutz

**Affiliations:** Clinical Laboratory, Vetsuisse Faculty, University of Zurich, Zurich, Switzerland; Section of Epidemiology, Vetsuisse Faculty, University of Zurich, Zurich, Switzerland

**Keywords:** Cat, Platelet count, Platelet aggregation, Deaggregation, Veterinary haematology, Feline, White blood cell count, Blood smear evaluation, Impedance analysers, Sysmex-XT 2000iV

## Abstract

**Background:**

Feline platelets are prone to clumping after blood collection, rendering the determination of accurate platelet counts difficult for clinical laboratories and resulting in a high incidence of pseudothrombocytopenia in feline haematology reports. No information is available about the kinetics of platelet aggregate formation in feline ethylenediaminetetraacetic acid blood and the course of platelet counts over a clinically relevant time period. The aim of the present study was to determine platelet counts in healthy cats over a time period of 24 h after blood collection at 9 time points; to assess potential effects of platelet aggregates, anaesthesia and bleeding conditions on feline platelets and white blood cell counts; and finally, to investigate if glucose concentration is associated with the presence of aggregates. From 30 clinically healthy cats, blood samples were analysed at 9 different time points using two different haematology instruments (using fluorescence and impedance-based flow cytometry) in the counting chamber and by blood smear evaluation.

**Results:**

Fourteen of the 30 samples were thrombocytopenic at one to 8 time points after collection as analysed on a fluorescence flow cytometry haematology analyser. At the 24-h timepoint, all thrombocytopenic samples had returned to normal platelet counts. Seventeen of the 30 samples showed platelet aggregates in the counting chamber. Significant differences in platelet counts were associated with the presence and size of aggregates and time since bleeding. No statistically significant differences in counts were found with regard to the quality of blood collection or the use of anaesthesia. Platelet aggregation and, therefore, pseudothrombocytopenia occurred in 57 % of the investigated samples at different time points.

**Conclusion:**

For the first time, deaggregation of feline platelet aggregates could be demonstrated as a reversible effect of platelet aggregation. For clinical laboratories or veterinarians, it may be helpful to rerun feline samples with pseudothrombocytopenia to obtain a more reliable platelet count. The quality of blood collection seems not to be causative for platelet aggregation. Blood smear evaluation is absolutely indicated in cases when haematology instruments give PLT counts below the reference interval.

**Electronic supplementary material:**

The online version of this article (doi:10.1186/s12917-015-0590-7) contains supplementary material, which is available to authorized users.

## Background

Feline platelets (PLT) are prone to clumping after blood collection, rendering the determination of accurate PLT counts difficult. In vitro aggregation of feline PLT; and, therefore, pseudothrombocytopenia is reported in 36 % [1], 62 % [2] and 71 % [3, 4] of feline EDTA samples. In contrast, true thrombocytopenia occurs rarely in cats; there are reports of 1.2 % [5] and 3.1 % [3] in EDTA samples of cats. Manual chamber counting is still accepted as the reference method for feline PLT counts in cats, despite the high degree of imprecision of this technique [6]. In impedance-based haematology instruments, which are widely used in veterinary practice, PLT counts are determined based on the volume of the platelets [7]. As the size of feline PLT often overlaps with the size of erythrocytes, PLT counts are inaccurate. Furthermore, impedance technique is highly affected by the presence of PLT clumps resulting in falsely increased white blood cell (WBC) counts [8]. Laboratory analysers using more sophisticated technologies are able to perform feline PLT counts based on fluorescence flow cytometry. The latest instrument, the Sysmex XT-2000iV (Sysmex Corporation, Kobe, Japan) showed very good agreement between the manual PLT count and the optical PLT count in feline EDTA samples without PLT aggregates [9]. Estimation of PLT count can be reliably obtained by examination of a stained blood smear in cats [10, 11]. Blood smear evaluation is mandatory to check for the presence of PLT aggregates. Several factors unique to feline PLT may be involved in their being prone to aggregate including a large PLT size, a higher concentration of serotonin, irreversible aggregation and granule release when exposed to serotonin, and irreversible aggregation in response to low concentrations of ADP [12]. It has been postulated that the quality of blood collection is the major cause for the presence of PLT aggregates in feline blood samples [1, 13]. Injury of the vessel endothelium caused by the needle used in blood collection leads to the adherence of PLT to subendothelial collagen-bound von Willebrand factor with the PLT GPIbα receptors inducing recruitment of further PLT [14]. Furthermore, PLT aggregation depends on adequate intracellular and extracellular energy sources. Intracellular glycogen stores and glucose are the major energy sources for ATP production in PLT. PLT are also able to take up glucose from the circulation through membrane glucose transporters [20]. Therefore, glucose concentration in the blood might have an impact on the formation and maintenance of PLT aggregates in the cat. To the authors’ knowledge no information is available about the kinetics of PLT aggregate formation in feline ethylenediaminetetraacetic acid (EDTA) blood samples, and the course of PLT counts over a clinical relevant time period of 24 h (h). Four different methods for PLT counting were used in the present study to counterbalance weaknesses of each individual technique. The aim of the present study was to determine PLT counts in healthy cats over a time period of 24 h at 9 time points, and to: (1) assess potential effects of PLT aggregates, anaesthesia (yes vs. no) and bleeding conditions (without any problem vs. difficulties) on feline platelet counts measured on a fluorescence flow cytometry-based instrument (Sysmex XT-2000iV) and an impedance-based haematology instrument (Mythic 18); (2) to describe potential effects of aggregates, anaesthesia and bleeding conditions on WBC counts measured by both technologies; and (3) to assess if glucose concentration is associated with the presence of aggregates. The results of the present study provide insights into the kinetics of feline PLT counts and influencing factors, which affect the results of laboratory diagnostic testing.

## Methods

### Blood samples

From 30 clinical healthy cats blood samples were obtained from the jugular vein. Blood collections were performed in accordance with Swiss law and were officially approved by the veterinary office of the canton of Zurich (TVB 100/2007, 101/2007, 99/2007). The cats were kept in groups under ethologically and hygienically ideal conditions, as described [15]. A completed ARRIVE guidelines checklist is included in Additional file 1. Blood was collected with a 22 G needle Neolus (Terumo, Leuven, Belgium) and a 5 mL Omnifix syringe (B. Braun, Melsungen, Germany). Immediately after collection, the needle was removed, and the blood was placed in a 4 mL K_3_- EDTA Vacuette tube (Greiner Bio-One GmbH, St. Gallen, Switzerland) and mixed gently for 2 min. In 18 of 30 cats, blood collection was performed under deep sedation (10 mg/kg Ketamine, Narketan, Vétoquinol AG, 0.1 mg/kg Midazolam, Dormicum, Roche Pharma AG, Rotkreuz, Switzerland). Blood collection was considered as “perfect” if the “first stitch” with the needle was in the vein and the blood was running well. Otherwise, blood collection was considered as “difficult” meaning a second stitch was necessary or blood flowed slowly and discontinuously. The quality of blood collection was perfect in 20 cats and difficult in 10 cats. The blood samples were analysed 30 min (min) after blood collection, and at each hour thereafter, until time point 7.5 h and finally at time point 24 h. Between these time points, the blood was stored at room temperature and mixed on an automated mixer (rock ‘n roller 34201, Snijders Scientific B.V.). At each time point, the blood samples were analysed consecutively on the Sysmex XT-2000iV (Sysmex Corporation, Kobe, Japan) and the Mythic 18 (Orphée SA, Geneva, Switzerland) for total WBC counts and PLT counts. Additionally, PLT counts were determined by manual chamber counting, and a blood smear was prepared. Glucose concentration was measured on the ACCU-CHEK glucometer (Roche Diagnostics GmbH, Rotkreuz, Switzerland).

### Instruments and methods used

The Sysmex XT-2000iV was used for total WBC and PLT counts [16]. WBC and PLT counts were analysed optically via a fluorescence flow cytometry method using a semiconductor laser. The Mythic 18 is an impedance-based haematology in-house analyser and has been validated for use in the cat [17]. PLT count was determined manually using a Neubauer improved hemocytometer (Assistent, Glaswarenfabrik Karl Hecht GmbH&Co KG) and a 1:100 dilution with 990 μl ThromboCount Pur (Bioanalytic GmbH). For each cat and each measuring time point, the entire counting chamber was examined for the presence of platelet aggregates. If platelet aggregates were found in the chamber, a qualitative PLT enumeration was not performed, instead a scoring schema was applied to describe the size of platelet aggregates. The score was established as followed: 0 = no aggregates; 1 = aggregates of 2–5 PLT; 2 = aggregates of 6–15 PLT; 3 = aggregates of 16–50 PLT; and 4 = aggregates >50 PLT. At each time point, a blood smear was prepared from each blood sample. Blood smears were stained using an automated staining instrument (HemaTek, Siemens). All blood smears were examined by one of the authors for the presence of PLT aggregates applying the same scoring system that had been used for the manual chamber counting technique. For the presence of aggregates a variable, which sums up the presence and size of aggregates (“gAgg” from 1 to 3), was used for the analysis. Cats showing no PLT aggregates over the observation period in the manual counting chamber were assigned to “gAgg1”. To assign a cat to “gAgg2” or “gAgg3”, results of the PLT aggregate score were added together and judged to be >1 to 10 (“gAgg2”), or >10 (“gAgg3”). Glucose concentration was measured hourly until time point 7.5 h.

### Statistical analysis

Statistical analyses were performed with the software packages R and nlme [18]. Linear mixed-effects models were performed to model the outcome variables PLT and WBC counts measured by the Sysmex XT-2000iV and the Mythic 18 instrument with the potential explanatory variables’ presence (and magnitude/importance) of aggregates (classified into the three groups of “no aggregates”, “low to moderate number of aggregates” and “high/important number of aggregates”), anaesthesia (yes or no), quality of blood collection (classified into “without any problem” and “difficult”) and the nine different time points. Model selection and validation was based on Akaike’s information criterion AIC and by checking visually the residuals for normality, homogeneity and independence. Significant differences were set at *p < 0.05*. For both instruments, the final models contained as explanatory variables the presence of aggregates, time, bleeding, anaesthesia, interaction between presence of aggregates and time, interaction between the presence of aggregates and bleeding as fixed effects and the individual cats as random effects. The random structure was extended using different variances for the groups of aggregates for the Sysmex XT-2000iV and different variances for the time points for the Mythic 18. For the time-correlated measurements, an AR-1 correlation structure was added. Linear mixed effects models were performed to model the outcome variable glucose by the potential explanatory variables of aggregates, time, anaesthesia and quality of blood collection. Glucose concentration under the detection limit (<0.6 mmol/l) of the instrument was set arbitrarily at 0.5 mmol/l. Bar charts were made using GraphPad Prism (version 3.00 for Windows, GraphPad Software).

## Results

### PLT

**Fig. 1:**
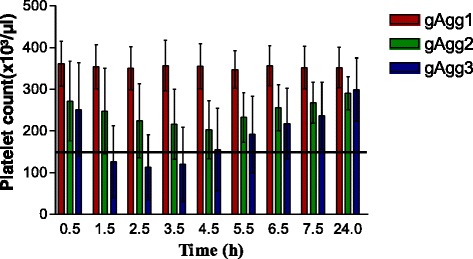
Bar chart for PLT counts of gAgg1 (red bar), gAgg2 (green bar), and gAgg3 (blue bar) determined by Sysmex XT-2000iV at 0.5 h, 1.5 h, 2.5 h, 3.5 h, 4.5 h, 5.5 h, 6.5 h, 7.5 h and 24 h after blood collection. The black line indicates the laboratory’s lower reference limit for feline platelets (180 × 10^3^/μL)

**Fig. 2:**
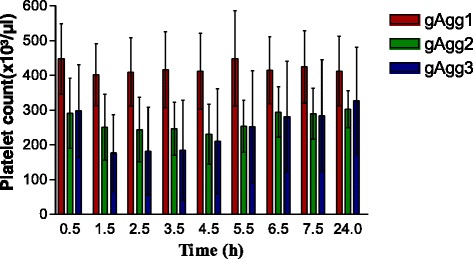
Bar chart for PLT counts of gAgg1 (red bar), gAgg2 (green bar), and gAgg3 (blue bar) determined by Mythic 18 at 0.5 h, 1.5 h, 2.5 h, 3.5 h, 4.5 h, 5.5 h, 6.5 h, 7.5 h and 24 h after blood collection. The black line indicates the laboratory’s lower reference limit for feline platelets (180 × 103/μL)

Fourteen of 30 samples analysed on the Sysmex XT-2000iV showed PLT counts under the lower reference interval (180–680 × 10^3^/μL) at a minimum of one time point and a maximum of 8 time points (Fig. 1). In 3 of the 14 samples with PLT aggregates, thrombocytopenia was detected already at the first time point (30 min after blood collection). In two of the 14 samples, thrombocytopenia occurred not before time point 4.5 h after blood collection. At time point 24 h, all samples were back within the reference range. PLT count analysis on the Mythic 18 showed the same trend as the results obtained from the Sysmex XT-2000iV; however, only 12 feline samples showed PLT counts under the lower reference range (Fig. 2).

With the manual chamber counting method, 17 feline samples with PLT aggregates were identified. The 13 cats without PLT aggregates were attributed to “gAgg1”. Seven cats with PLT aggregates were attributed to “gAgg2”. PLT aggregates of these seven cats showed a maximal score of 2. The remaining 10 cats with PLT aggregates belonged to “gAgg3”. At the time point of 24 h, 3 samples still showed PLT aggregates in the counting chamber.

Mean and standard errors (std.error) for PLT counts measured by the Sysmex XT-2000iV and by the Mythic 18 instrument with the potential explanatory variables presence of aggregates (gAgg), anaesthesia, quality of blood collection and the different time points are presented in Table 1. Significant differences in PLT counts were associated with presence and size of aggregates (Sysmex XT-2000iV: p = 0.001*; Mythic 18: p = 0.039*) and time (Sysmex XT-2000iV: p = 0.029*; Mythic 18: p = 0.147*) after bleeding. The increase in presence and size of the aggregates was found to be associated with a decrease in PLT of 86.1 × 10^3^/μL (std. error 23.81 × 10^3^/μL). Additionally a significant interaction between aggregates and time was present (Sysmex XT-2000iV: p = 0.034*; Mythic 18: p < 0.001*) indicating that the course of PLT counts differed with the presence and size of aggregates. The Mythic 18 device is similar to the Sysmex XT-2000iV device with regard to the variables of presence of aggregates and interaction between aggregates and time. However, in contrast to the Sysmex XT-2000iV device, time is not associated with significant differences. The estimated group effect of aggregates is −95.68 × 10^3^/μL (std. error 43.93 × 10^3^/μl). No statistical significance was found for quality of blood collection (Sysmex XT-2000iV: *p = 0.189*; Mythic 18: *p = 0.112*), anaesthesia (Sysmex XT-2000iV: *p = 0.654*; Mythic 18: *p = 0.405*) and the interaction between presence of aggregates and quality of blood collection (Sysmex XT-2000iV: *p = 0.513*; Mythic 18: *p = 0.082*).

**Table 1:** Mean and standard errors (std.error) for PLT counts measured by the Sysmex XT-2000iV and the Mythic 18 instruments, with the potential explanatory variables being the presence of aggregates (gAgg), anaesthesia, quality of blood collection and the different time points

Variable		Sysmex XT-2000iV PLT (x10^3^/μL); mean ± std.error	Mythic 18 PLT (x103/μL); mean ± std.error
gAgg	gAgg1	354.2 ± 52.2	421.0 ± 107.5
	gAgg2	245.7 ± 79.9	267.3 ± 86.3
	gAgg3	190.3 ± 109.3	244.0 ± 156.0
Time	0.5 h	303.9 ± 102.5	361.2 ± 138.2
	1.5 h	253.4 ± 128.5	291.4 ± 142.7
	2.5 h	242.3 ± 127.8	295.1 ± 151.5
	3.5 h	245.2 ± 131.5	299.7 ± 158.9
	4.5 h	253.2 ± 119.8	302.7 ± 156.6
	5.5 h	268.9 ± 98.9	337.6 ± 168.8
	6.5 h	286.9 ± 91.0	342.4 ± 135.5
	7.5 h	294.1 ± 82.6	346.6 ± 141.5
	24 h	320.2 ± 65.2	358.3 ± 125.7
Quality of blood collection	difficult	239.1 ± 108.4	284.1 ± 149.7
	perfect	291.8 ± 105.7	347.1 ± 142.1
Anaesthesia	Yes	274.2 ± 118.4	326.6 ± 167.5
	no	274.2 ± 94.3	325.4 ± 111.3

### WBC

**Fig. 3:**
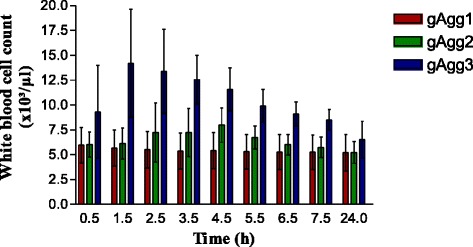
Bar chart for WBC counts of gAgg1 (red bar), gAgg2 (green bar), and gAgg3 (blue bar) determined by Mythic 18 at 0.5 h, 1.5 h, 2.5 h, 3.5 h, 4.5 h, 5.5 h, 6.5 h, 7.5 h and 24 h after blood collection

Mean and standard errors (std.error) for WBC counts measured by the Sysmex XT-2000iV and the Mythic 18 instrument with the potential explanatory variables of presence of aggregates (gAgg) and of the different time points are presented in Table 2 and Fig. 3 (Mythic 18). Significant differences in WBC counts are associated with presence and magnitude of aggregates in the impedance-based haematology analyser (Mythic 18: p = 0.002*) with an estimated group effect of 1.65 × 10^3^/μL (std. error 0.73 × 10^3^/μL) but not in the fluorescence flow cytometry-based instrument (Sysmex XT-2000iV: *p = 0.283*). Measurements from both instruments are significantly lower in cats with anaesthesia (Sysmex XT-2000iV: p < 0.001*; Mythic 18: p < 0.003*) with an estimated effect of anaesthesia of −2.11 × 10^3^/μL (std .error 0.65 × 10^3^/μL), WBC for Mythic 18 and −3.44 × 10^3^/μL (std. error 0.72 × 10^3^/μL) and WBC for Sysmex XT-2000iV. WBC counts determined by both instruments were significantly influenced by the time period (Sysmex XT-2000iV: p < 0.001*; Mythic 18: p < 0.001*); however, differences were small and without clinical relevance. WBC counts determined in the optical channel of the Sysmex XT-2000iV showed no significant changes due to PLT aggregates. In 10 cats, WBC counts determined by the Mythic 18 showed an increase in total WBC counts at different time points over the examination period from normal values to leukocytotic WBC counts, which came back to normal values no later than the time point of 24 h. In all ten cats, PLT aggregates have been identified in the manual counting chamber, and 9 out of 10 cats belonged to “gAgg3”, one cat to “gAgg2”. Additionally, 2 cats with leukopenic WBC counts at time point 30 min had temporarily normal WBC counts at a later time point, and reached leukopenic values at time point 24 h. In two other cases, WBCs increased by 3000 and 5000/μL, but within the reference interval (4.6-12.8 × 10^3^/μL). At time point 24 h, ten feline samples showed normal WBC counts.

**Table 2:** Mean and standard errors (std.error) for white blood cell (WBC) counts measured by the Sysmex XT-2000iV and the Mythic 18 instruments, with the potential explanatory variables being the presence of aggregates (gAgg) and the different time points

Variable		Sysmex XT-2000iV WBC (x10^3^/μL); mean ± std.error	Mythic 18 WBC (x103/μL); mean ± std.error
gAgg	gAgg1	7.0 ± 2.5	5.4 ± 1.8
	gAgg2	6.7 ± 1.7	6.5 ± 1.9
	gAgg3	8.5 ± 2.7	10.6 ± 4.0
Time	0.5 h	7.7 ± 2.6	7.1 ± 3.4
	1.5 h	7.6 ± 2.6	8.6 ± 5.3
	2.5 h	7.5 ± 2.6	8.5 ± 4.7
	3.5 h	7.5 ± 2.6	8.2 ± 3.9
	4.5 h	7.4 ± 2.6	8.1 ± 3.3
	5.5 h	7.4 ± 2.6	7.2 ± 2.6
	6.5 h	7.4 ± 2.6	6.7 ± 2.3
	7.5 h	7.4 ± 2.6	6.5 ± 2.1
	24 h	7.1 ± 2.5	5.7 ± 1.8

### Glucose

Significant differences in glucose levels were associated with the presence and magnitude of aggregates (*p* = 0.014*; estimated effect of glucose of −0.33 mmol/L, std .error 0.13 mmol/L) and time after bleeding (*p* < 0.001; estimated effect of glucose of −0.34 mmol/L, std .error 0.01 mmol/L). Glucose concentration in EDTA whole blood ranged at the first measurement time point from 2.7 to 6.3 mmol/L. One cat at time point 5.5 h, two cats at time point 6.5 h, and four cats at time point 7.5 h showed glucose concentrations under the detection limit of the instrument. The latter cats belong to “gAgg3”.

## Discussion

This is the first study on feline EDTA blood demonstrating a time dependent effect on the formation and deaggregation of PLT aggregates. In 47 % of the investigated cats, a significant decrease of PLT counts under the lower reference limit was observed with the Sysmex XT-2000iV instrument. In parallel, PLT aggregates were identified in the counting chamber and in the blood smear. At the last measuring time point, all cats had PLT counts within the reference interval with the Sysmex XT-2000iV, whereas in the counting chamber and the blood smear, PLT aggregates were smaller (scoring index decreased) or disappeared. Based on the observations, it is very likely that in feline EDTA blood, PLT aggregation occurs at different time points after blood collection, and, furthermore, PLT aggregates dissolve in the course of time. The first occurrence, the duration, and the deaggregation of PLT aggregates took place at different time points in each individual cat. Consequently, 47 % of the cats in this study were identified with false positive thrombocytopenia (pseudothrombocytopenia) when relying only on the electronic PLT count. Six out of the 14 cats showed severe thrombocytopenia, with PLT counts below 50 × 10^3^/μL. This observation is of great clinical relevance for the determination of feline PLT counts in veterinary haematology. Blood samples are usually analysed at different time points after collection depending on if in-house analysis or external laboratories are chosen. Additionally, the time of the first occurrence of PLT aggregates differed among individual cats. Based on the results of this study, no recommendation concerning the best analysis time point for feline PLT counts can be given. If the time point of PLT analysis matches with PLT aggregation, blood smear evaluation or manual chamber counting is indicated to differentiate between true thrombocytopenia and pseudothrombocytopenia.

It was reported that the quality of blood collection is the major cause of PLT aggregates in feline EDTA blood [1, 12, 13]. Small vessels and difficulties in handling of the feline often result in a difficult blood collection. In the present study the quality of blood collection had no statistically significant influence on the electronically determined PLT counts and, therefore, on the presence of PLT aggregates. In addition, no statistically significant influence of anaesthesia on PLT counts was observed. Moreover, perfect blood collection did not prevent PLT aggregation in the present study.

Clinical laboratories or veterinarians are advised to determine PLT counts within a relatively short time span after blood collection. Moritz et al. recommended PLT counting within 30 min after blood collection [1], Weiser et al. postulated counting PLT within 1 h [13], and Knoll et al. recommended counting PLT 4 h to 6 h after blood collection [8]. In the present study, PLT aggregation and, therefore, low PLT counts already occurred at the time point of 30 min after collection, and even at time point 6.5 h PLT aggregates were present. At time point 24 h after collection, Sysmex XT-2000iV PLT counts were within the reference intervals in all investigated feline samples, and correlated quite well with the PLT counts at time point 30 min in cats without PLT aggregates. Therefore, it is very likely that, in the presence of PLT aggregates, PLT counts at time point 24 h give the most reliable result.

The peculiarity of feline PLT to be more reactive than PLT from other species paired with a high likelihood of building aggregates are well-known phenomena and have been described extensively in the literature. It has been assumed that PLT aggregation in feline blood samples is irreversible in response to low concentrations of ADP [12]. In contrast, Hart and Nolte [19] demonstrated that ADP concentrations ≤ 0.8 μM induced a reversible aggregation, whereas ADP concentration between 1–2 μM induces an irreversible PLT aggregation. The present study demonstrates that the aggregation response in feline EDTA samples includes a reversible component. In all feline samples with PLT aggregates, deaggregation has been observed in the manual chamber counting and the blood smear. In parallel, impedance and optical PLT values increased, and came back to normal PLT values. It could be observed that PLT from samples that built aggregates were generally paler than the PLT in samples without PLT aggregation, suggesting that aggregation led to degranulation, whereas single PLT retained their granules. Formation and maintenance of PLT aggregations depends on adequate intracellular and extracellular energy sources. Intracellular glycogen stores and glucose are the major energy sources for ATP production in PLT. PLT are also able to take up glucose from the circulation through membrane glucose transporters [20]. Therefore, it can be speculated that deaggregation might occur due to an intracellular energy deficiency in degranulated PLT. Furthermore, measurement of peripheral blood glucose concentration showed not only a significant decrease over time but also a significant influence of PLT aggregates with glucose concentration. Deaggregation of human PLT is known to be an integral component of the response of PLT to ADP in vitro [21]. Further studies are needed to investigate the mechanism of feline PLT deaggregation.

The Sysmex XT-2000iV has the ability to count feline PLTs through the use of a fluorescent nucleic acid dye, overcoming the problem of size-overlapping between RBCs and PLTs in impedance-based instruments [16]. It therefore provides the latest technology for feline PLT count in a routine clinical laboratory. However, the diluent and the sheath fluid of the Sysmex XT-2000iV are not able to dissolve feline PLT aggregates and count them as single PLT. Furthermore, no error message for the optical PLT had been reported by the instrument. Optical cell counting methods are not able to count aggregates, because their light scatter pattern is different from individual PLT [2].

**Fig. 4:**
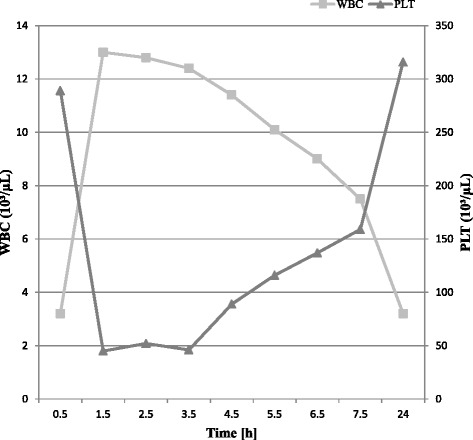
Line chart for WBC counts (primary y-axis, light grey line, and squares) and PLT count (secondary y-axis, grey line, and triangles) of a feline EDTA sample with PLT aggregates determined by Mythic 18 over 0.5 h, 1.5 h, 2.5 h, 3.5 h, 4.5 h, 5.5 h, 6.5 h, 7.5 h and 24 h after blood collection (x-axis)

In addition, an impedance-based haematology instrument for in-house analysis has been used in this study to demonstrate the influence of PLT aggregates. In contrast to the Sysmex XT-2000iV, PLT aggregates showed a statistically significant influence on WBC counts released by the Mythic 18. Ten cats with PLT aggregates showed false positive leukocytosis. It is a well-known phenomenon in cats that platelet clumps cause falsely increased WBC count and decreased PLT counts in impedance-based haematological instruments [3, 8]. From our observations, it can be concluded that in impedance-based haematology instruments, the decrease of PLT counts and the increase of WBC counts due to PLT aggregates occurs reciprocally (Fig. 4).

Cats that had blood collection without anaesthesia showed significantly higher WBC counts compared to cats that had blood was drawn under anaesthesia. As all cats used in this study were clinically healthy, the difference in WBC counts can be mainly attributed to stress in those cats without anaesthesia resulting in higher WBC counts [22].

## Conclusions

This is the first study to observe feline PLT counts in EDTA blood samples over a time course of 24 h with respect to the presence of PLT aggregates. Aggregation occurred in 57 % of the investigated samples at different time points and with different frequencies. The quality of blood collection seems not to be causative for PLT aggregation. For the first time, deaggregation of feline PLT aggregates could be demonstrated as a reversible effect of aggregation. For clinical laboratories or veterinarians, it might be helpful to rerun feline samples with pseudothrombocytopenia due to PLT aggregates after several hours to obtain a more reliable PLT count. Generally, blood smear evaluation or manual chamber counting is indicated in cases where haematology instruments give PLT numbers below the reference interval.

## Additional file

Additional file 1:
**Completed "The ARRIVE Guidelines Checklist" for reporting animal data in this manuscript.** (DOCX 660 kb)

## Abbreviations

EDTA: ethylenediaminetetraacetic acid
h: hours
PLT: platelets
min: minutes
WBC: white blood cells
