# Supplementary material for: Study on the kinetics and influence of feline platelet aggregation and deaggregation
Source: BMC Vet Res. 2015 Nov 5;11:276. doi: 10.1186/s12917-015-0590-7 (PMC4635602; doi:10.1186/s12917-015-0590-7)

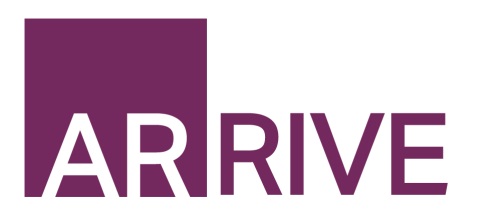


The ARRIVE Guidelines Checklist

Animal Research: Reporting In Vivo Experiments

Carol Kilkenny^1^, William J Browne^2^, Innes C Cuthill^3^, Michael Emerson^4^ and Douglas G Altman^5^

*^1^The National Centre for the Replacement, Refinement and Reduction of Animals in Research, London, UK, ^2^School of Veterinary Science, University of Bristol, Bristol, UK, ^3^School of Biological Sciences, University of Bristol, Bristol, UK, ^4^National Heart and Lung Institute, Imperial College London, UK, ^5^Centre for Statistics in Medicine, University of Oxford, Oxford, UK.*

|  | | ITEM | RECOMMENDATION | Section/ Paragraph |
| --- | --- | --- | --- | --- |
| 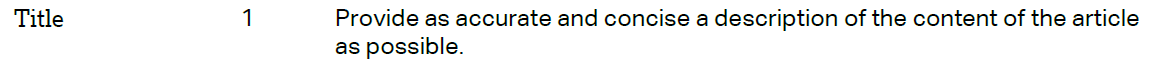 | | | Title |  |
| 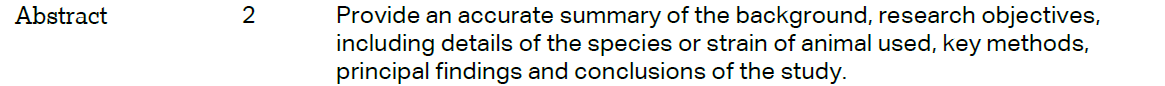 | | | Abstract |  |
| INTRODUCTION | | |  |  |
| 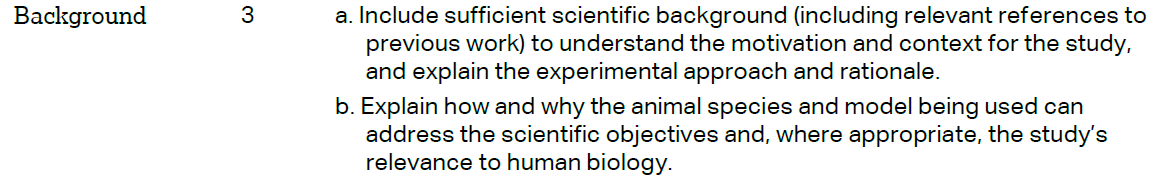 | | | Introduction |  |
| 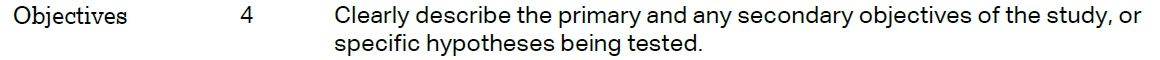 | | | Introduction (last paragraph) |  |
| METHODS | | |  |  |
| 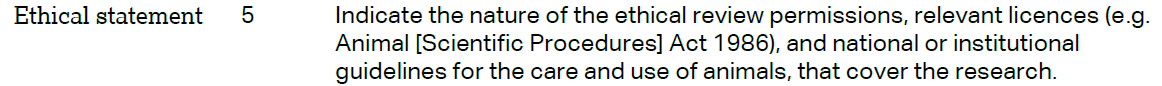 | | | Methods (Blood samples) |  |
| 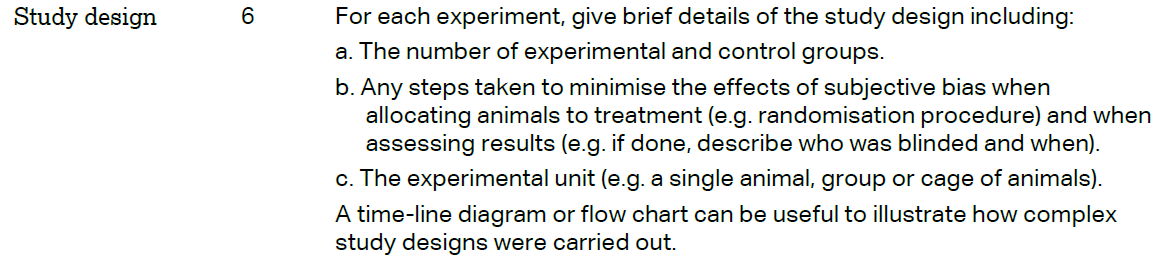 | | | Methods (Blood samples) |  |
| 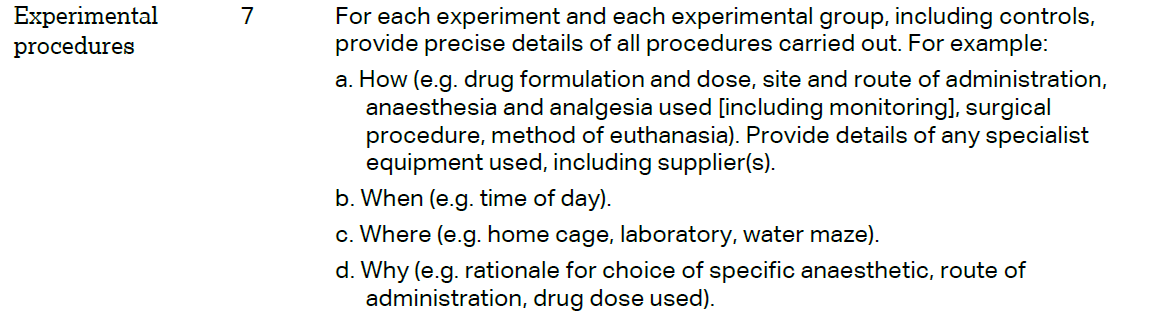 | | | Methods (Blood samples) |  |
| 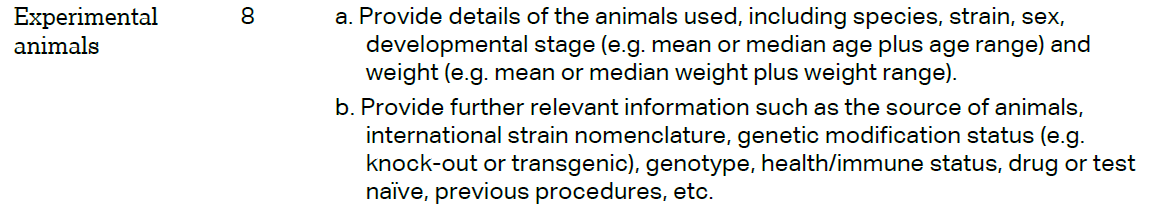 | | | Methods (Blood samples) |  |

The ARRIVE guidelines. Originally published in *PLoS Biology*, June 2010^1^

| 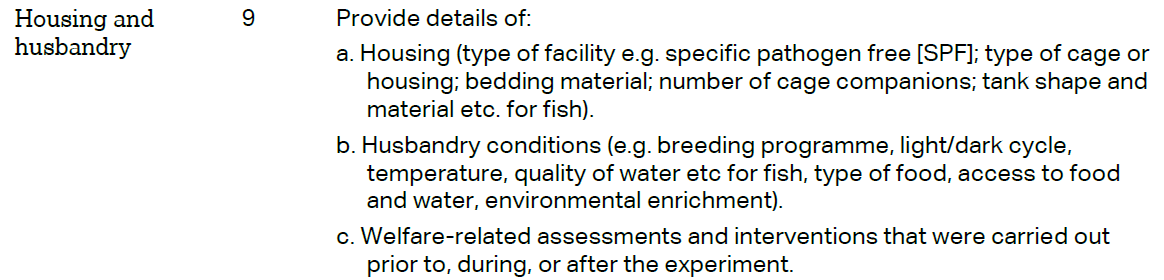 | Methods (Blood samples) | |
| --- | --- | --- |
| 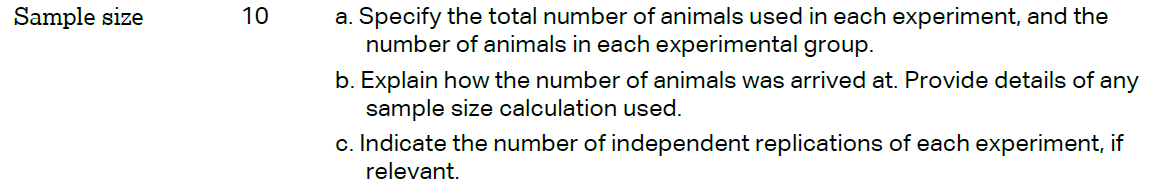 | Methods (Blood samples) | |
| 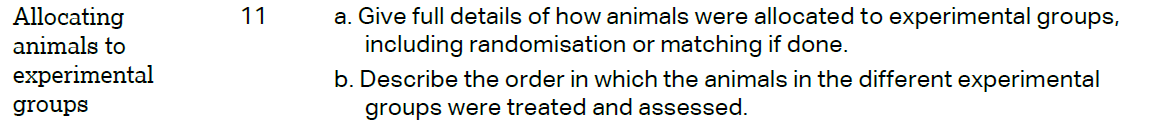 | - | |
| 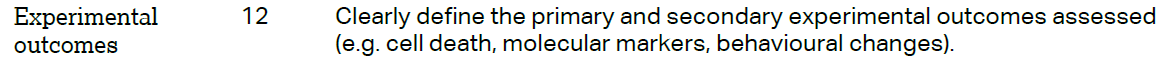 | Paragraph “Results” | |
| 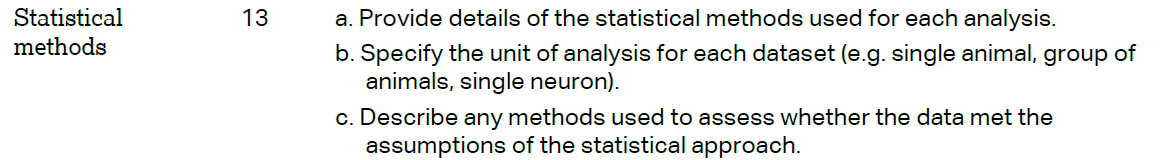 | Paragraph “Statistical analysis” | |
| RESULTS |  | |
| 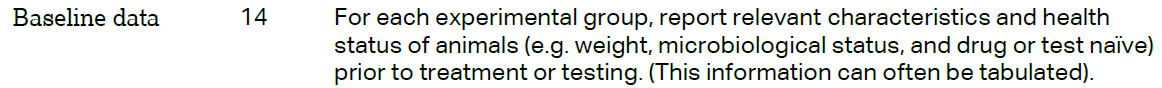 | - | |
| 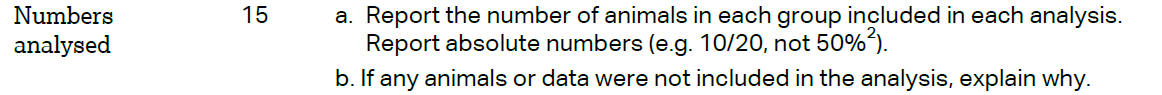 | - | |
| 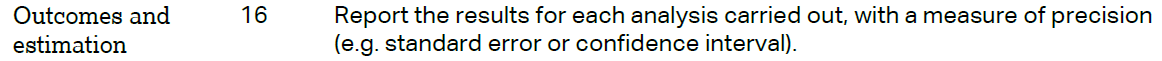 | Paragraph “Results” and Table 1 and 2 and Figure 1-4 | |
| 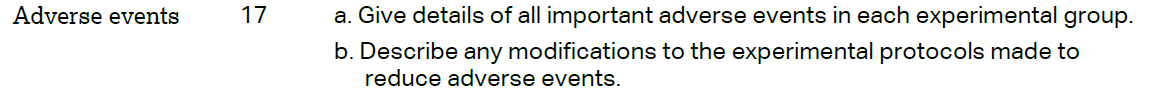 | - | |
| DISCUSSION |  | |
| 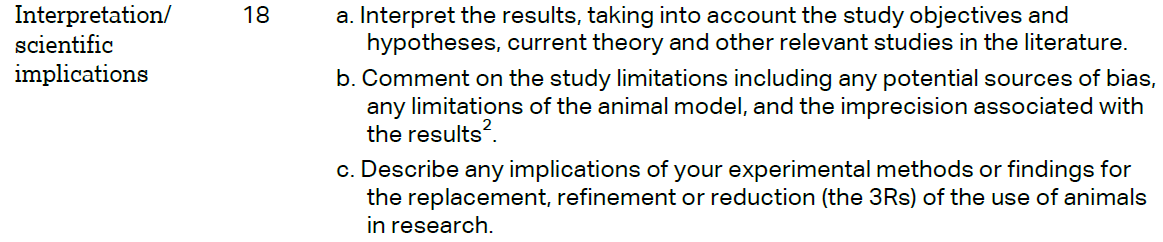 | Discussion | |
| 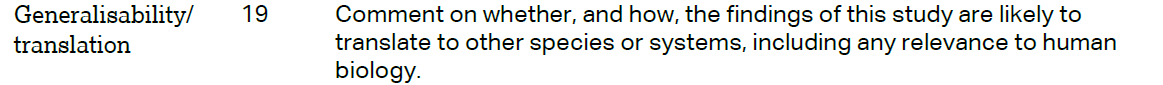 | Discussion/Conclusion | |
| 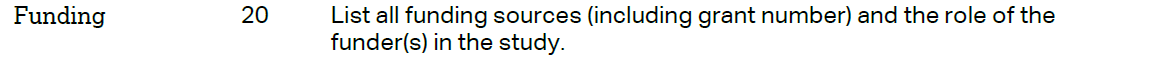 | | - |


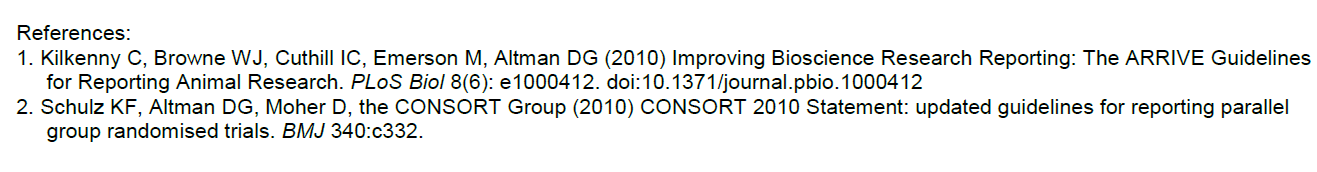

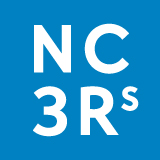

Supplement: Additional file 1: — Completed "The ARRIVE Guidelines Checklist" for reporting animal data in this manuscript. (DOCX 660 kb) [file 12917_2015_590_MOESM1_ESM.docx]
